# Supplementary material for: COZOID: contact zone identifier for visual analysis of protein-protein interactions
Source: BMC Bioinformatics. 2018 Apr 6;19:125. doi: 10.1186/s12859-018-2113-6 (PMC5889581; doi:10.1186/s12859-018-2113-6)
Supplement: Supplementary file 2 — Software build. Executable binary file of the software tool. (ZIP 118,784 kb) [file 12859_2018_2113_MOESM2_ESM.zip › cozoid/java/docs/beansbinding-1.2.1-doc.zip!/org/jdesktop/el/Expression.ResolvedProperty.html]

Expression.ResolvedProperty (Beans Binding)


---


|  |  |  |
| --- | --- | --- |
| **Overview** \| **Package** \| **Class** \| **Use** \| **Tree** \| **Deprecated** \| **Index** \| **Help** | |  |
| **PREV CLASS**   **NEXT CLASS** | **FRAMES**    **NO FRAMES**     **All Classes** |  |
| SUMMARY: NESTED \| FIELD \| CONSTR \| METHOD | DETAIL: FIELD \| CONSTR \| METHOD |  |


---


## org.jdesktop.el Class Expression.ResolvedProperty

```
java.lang.Object
  org.jdesktop.el.Expression.ResolvedProperty
```

**Enclosing class:**: Expression

---

``` public static final class Expression.ResolvedProperty extends java.lang.Object ```

---

**Constructor Summary**

`Expression.ResolvedProperty(java.lang.Object source,
java.lang.Object property)`


| **Method Summary** | |
| --- | --- |
| `boolean` | `equals(java.lang.Object o)` |
| `java.lang.Object` | `getProperty()` |
| `java.lang.Object` | `getSource()` |
| `int` | `hashCode()` |

**Methods inherited from class java.lang.Object**

`clone, finalize, getClass, notify, notifyAll, toString, wait, wait, wait`

**Constructor Detail**

### Expression.ResolvedProperty

```
public Expression.ResolvedProperty(java.lang.Object source,
                                   java.lang.Object property)
```


**Method Detail**

### getSource

```
public java.lang.Object getSource()
```

---


### getProperty

```
public java.lang.Object getProperty()
```

---


### equals

```
public boolean equals(java.lang.Object o)
```

:   **Overrides:**: `equals` in class `java.lang.Object`

---


### hashCode

```
public int hashCode()
```

:   **Overrides:**: `hashCode` in class `java.lang.Object`


---


|  |  |  |
| --- | --- | --- |
| **Overview** \| **Package** \| **Class** \| **Use** \| **Tree** \| **Deprecated** \| **Index** \| **Help** | |  |
| **PREV CLASS**   **NEXT CLASS** | **FRAMES**    **NO FRAMES**     **All Classes** |  |
| SUMMARY: NESTED \| FIELD \| CONSTR \| METHOD | DETAIL: FIELD \| CONSTR \| METHOD |  |


---
